# An empirical link between motivation gain and NBA statistics: applying hierarchical linear modelling

**DOI:** 10.1186/s40359-023-01188-1

**Published:** 2023-04-27

**Authors:** Yunsik Shim, Myoungjin Shin

**Affiliations:** 1 Department of Sports Science, Soonchunhyang University, Asan, South Korea; 2 Department of Leisure Sports, Kangwon National University, Samcheok, South Korea

**Keywords:** Motivation gain, Salaries, Köhler effect, Social compensation, NBA

## Abstract

**Background:**

This study tested whether the motivation gain in groups is the result of social compensation or the Köhler effect by examining scaled individual salaries of National Basketball Association (NBA) players. Both factors explain the positive effects of a group, unlike social loafing. However, differing causes in motivation gain relate to whether players are low or high performers and the Köhler effect or social compensation.

**Methods:**

To test motivation gain, this study used 11-year NBA statistical data of 3247 players by applying hierarchical linear modelling (HLM) and HLM 7.0 was used for analysis. The players’ individual statistics and annual salaries were collected from the NBA and ESPN websites, respectively. Whereas previous studies have looked at motivation gain through track-and-field and swimming relay records, this study verified motivation gain through salary variations among NBA players and their affiliated teams.

**Results:**

The high performers, while selecting teams with larger performance gaps among team members, earned a higher salary than while selecting teams with lower performance gaps among team members. This study found that motivation gain existed in high performers, which can be interpreted as support for social compensation rather than the Köhler effect.

**Conclusions:**

We used our result to elucidate the basis for play-by-play decisions made by individuals and team behaviour. Our results are applicable for the enhancement of coaching strategies, ultimately improving team morale and performance. It can be interpreted that the motivation gains of high performers in the NBA are driven by the *Cost Component* of the Team member Effort Expenditure Model (TEEM), rather than the *Expectancy and Value Components.*

## Background

Studies that have examined motivation gain in general either focused on laboratory tasks or real sports teams [1–6]. Contemporary researchers continue to examine this field and other related areas. Motivation gain studies that focused on sports used archival data either in track and field [5, 6] or swimming [1–4, 7], alone. In relay sports, such as track and field or swimming, motivation gain can be verified using the difference between individual and relay records. In sports that promote performance through interaction and cooperation among team members, however, how can the existence of motivation gain be confirmed? Additionally, does motivation gain exist in team sports outside those involving relay? In this study, after introducing a new approach to confirm motivation gain in the National Basketball Association (NBA) through the relationship between individual annual salary, objective performance indicators, and the standard deviation of team annual salary, the existence of motivation gain in the NBA was also confirmed.

### Motivation gain and loss in the NBA

Karau and Williams [8] introduced the Collective Effort Model (CEM) which is an integrative model of individual motivation within the group, to generate predictions of group outcomes based on individual efforts. The CEM assumes that an individual member’s effort in the course of teamwork is a function of three psychological factors, namely *Expectancy* (i.e. high levels of effort will lead to high levels of performance), *Instrumentality* (i.e. high-quality performance is instrumental in obtaining an outcome; [8], p. 685), and *Valence* (i.e. it is present if the outcomes of the performance are considered desirable; [9]). Karau and Williams [11] determined that expectancy multiplied by instrumentality multiplied by valence produces the resulting motivational force. Therefore, if any of the three factors converges to ‘0’, motivational force disappears, and the phenomenon of social loafing may exist.

Social loafing is a phenomenon wherein people invest lower efforts to achieve goals while working together than while working alone [10]. In CEM, members who participate in collective tasks invest a lot of effort when they believe that individual effort plays an important role in achieving group goals. However, when they do not do so, social loafing tends to be induced [11]. Hüffmeier and Hertel [12] presented two cases in which individual efforts can be elicited in collaborative tasks. The first is when individual members perceive outcomes related to the group’s performance as valuable, and the second is when better individual outcomes are expected because of one’s efforts while working collectively than while working individually.

By revenue, the NBA is the third wealthiest professional sport league in North America after the National Football League (NFL) and Major League Baseball (MLB), and ranks among the top four leagues in the world [13]. As of 2021, NBA players are the world’s best paid athletes by per-player average annual salary [14]. In the case of world-class professional leagues like the NBA, where only a few players are selected through fierce competition to play in the dream league, players gain fame and accumulate wealth through team wins and good performances. Athletes belonging to 30 NBA teams play 82 games during the regular season, trying to win as many as it takes to advance to the playoffs. Therefore, the possibility of social loafing in the NBA is low.

### Köhler effect and social compensation

Among various theoretical approaches, both the Köhler effect and social compensation induce motivation gain from opposing subjects. Both theories explain the motivation gain of team members, however, it may be induced differently depending on the member’s performance level. The former [15, 16] is a phenomenon in which effort is increased in low performers while performing a collective task with high performers [17], whereas the latter displays motivation gain among high performers [18]. In the Köhler effect, the motivation gain depends on the lower performer’s perceived social indispensability to the group, the discrepancy between the higher performers and themselves (social comparison), and avoidance of social stigma (impression management). These mechanisms are more relevant in conjunctive tasks and when the performance discrepancy is moderate. On the contrary, in social compensation effects, task importance, expected lower ability of fellow teammates, and disjunctive type tasks are more relevant mechanisms in explaining social compensation effects.

Many experimental studies have examined the Köhler motivation gain and have found consistent results for conjunctive tasks [19–24]. Summarising the results of these studies, productivity of the low performer improved under the conjunctive task condition in which team performance was determined by the low-performing member. Osborn et al. [4] investigated whether the Köhler effect exists in real-world sports settings by examining records of a relay swimming competition and an additive task in which a group competes by aggregating individual members’ efforts or contributions. They found a Köhler motivation gain where the low performer’s relay record was improved from their individual time. In the social compensation context, people put in greater effort in the collective setting than in the individual setting, one of the factors being deficient performance by a co-worker (such as a low performer). When a low performer’s contribution is insufficient, an individual (a high performer) must exert greater effort to achieve the collective goal [25, 26]. ‘Social compensation’ refers to the phenomenon wherein motivation gain occurs among high performers instead of low ones [18].

### Köhler effect and social compensation in the team member effort expenditure model

While CEM, with its emphasis on three psychological factors, has merit in explaining the emergence and reduction of effort loss in teams, it is not adequate as a theoretical framework to integrate the entire spectrum from motivation gain to motivation loss [27]. Torka et al. [27] presented the Team member Effort Expenditure Model (TEEM), which clearly explains not only the causes of effort loss, but also the causes of effort gain based on the studies of Karau and Williams [8] and Shepperd [28] TEEM explains that effort gain and effort loss can be caused by three components (1. *Expectancy*, 2. *Value*, and 3. *Cost*).

The *Expectancy Component*, which triggers effort gain, manifests as motivation gain when the behavior and outcome is perceived to be strongly related during teamwork. (e.g., In a swimming relay race (teamwork), the team members, except the last swimmer, have the expectation that even if his or her record is not good, the other team members will do well, and the team record will improve (low behavior and outcome relation)). However, the last player has no such expectations, so perceives that his or her performance has the greatest impact on the team’s performance (high behaviour and outcome relation), inducing effort gain [29]. Effort loss occurs when behavior and outcome is perceived to be weakly related in team work. (e.g., dispensability of individual contributions (free riding)) [30].

The *Value Component*, which triggers effort gain, occurs when the value of an action and/or outcome is perceived to be higher in teamwork than in individual work. (e.g., when a player can compare their performance to that of a moderately superior fellow member, they receive social comparison information that they wouldn’t have gotten in individual work and judge their efforts to be more valuable [31]. Effort loss occurs when the value of an action and/or outcome is perceived to be lower in teamwork than in individual work (e.g., nonidentifiability of contributions (social loafing)) [32].

Finally, the *Cost Component* causes effort gains when the cost/benefit ratio of effort expenditure is perceived more favorably in teamwork, than in individual work. (e.g., Stronger team members expend more effort because they believe that the abilities and efforts of weaker team members are insufficient to achieve the common goal [33]. Effort loss occurs when the cost/benefit ratio of effort expenditure is perceived to be less favorable in teamwork than in individual work (effort reduction as a response to free-riding fellow members (sucker effect)) [34].

Based on TEEM, the components that affect motivation gain in the NBA are value and cost. Since the NBA consists of the top players in the world, if the assumption that the performance gap between high and low performers is moderate is satisfied, the effort gains of low performers (Köhler effect) can be attributed to the *Value Component*. On the other hand, if the effort gains are present among high performers (social compensation), it can be concluded that the motivation gain is triggered by the *Cost Component*.

### Limitations of prior research and strengths of this study

Motivation gain-related studies have been conducted on real sports teams including track and field [5] and swimming [1–4]. Motivation gain in both these sports can be examined by comparing individual and relay records. However, there are some limitations. First, differences in the starting process between relay swimmers may affect individual performance. For instance, in swimming, whereas the first swimmer starts with a gun start, the others start with a flying start. In the latter, the swimmer can start based on the prediction that the front swimmer’s hand will make contact with the touch panel, so the reaction time is faster than that in a gun start. Hüffmeier et al. [1] adjusted the reaction time to offset the individual and relay performance errors induced by gun and flying starts. However, it is still difficult to see that the performance error because of the difference in the starting process is completely controlled. Second, additive tasks are not limited to swimming and track and field relays alone. Thus, motivation gains must be confirmed in a range of other sports as well. Soccer, basketball, and baseball are team sports in which a team strives to achieve a common goal while maintaining a collective relationship through interdependence and interactions among team members. Thus, it is also necessary to study motivation gain in cooperative sports and not just in additive tasks alone.

In this study, motivation gain was examined through the difference among the salaries of NBA players and teams. As an athlete’s salary can increase with an objective indicator of the overall evaluation of individual ability [35–37], it should be associated with other performance indices such as points (PTS) and efficiency (EFF). If the relationship between individual salaries and performance stats (e.g. PTS, EFF) is affected by a deviation in team salary (e.g. a team with a large team salary deviation comprises high and low performers, and while a team with a small team salary deviation comprises players with similar performances), it is possible to indirectly confirm motivation gain. This approach can contribute towards adopting a new method of verifying motivation gain that is completely different from the existing one that compares individual and relay records in track and field and swimming.

### Research purpose and hypotheses

**Fig. 1:**
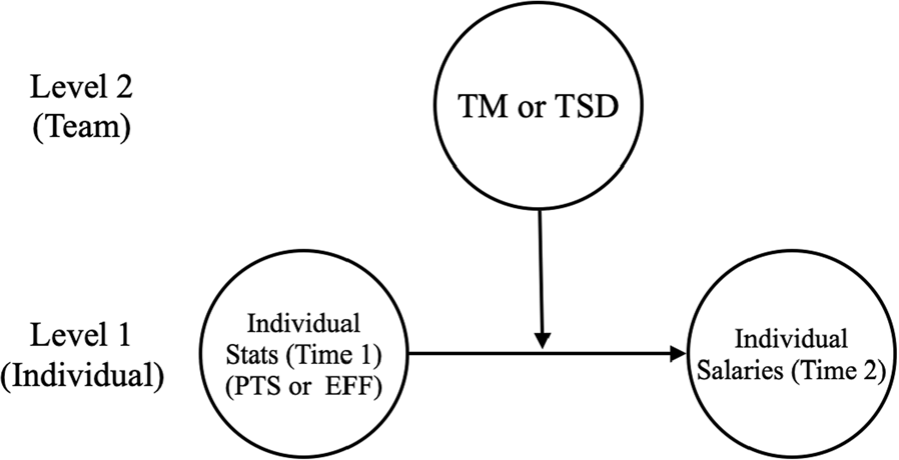
Research model. *Note* Time 1 = previous season. Time 2 = current season. PTS = points. EFF = efficiency. TM = total mean across 11 years of the mean salary for each team; TSD = total mean across 11 years of the standard deviation of salary for each team

This study tested whether the motivation gain in the NBA is the result of *Cost Component* (social compensation) or *Value Component* (Köhler effect) by examining scaled individual salaries of NBA players and standard deviations among the salaries in each team using hierarchical linear modelling (HLM) analysis. As shown in Fig. 1, HLM confirms how the slope of the previous season’s individual (level 1) stats (PTS or EFF) on the current season’s individual salaries is affected by team variables (level 2) such as TM (Total mean across 11 years of Mean salary for each team) or TSD (Total mean across 11 years of Standard Deviation of salary for each team).

Hypothesis 1 (supporting *Value Component* of TEEM [31]): The positive relation of individual PTS on individual salaries will be weaker for high performers when compared to low ones (Level 1), and this relationship will be found in teams with large standard deviations in salary, but not in those with high average salaries (Level 2). Hypothesis 2 (supporting *Value Component* of TEEM [31]): The positive relation of individual EFF on individual salaries will be weaker for high performers when compared to low ones (Level 1), and this relationship will be found in teams with large standard deviations in salary, but not in those with high average salaries (Level 2). Hypothesis 3 (supporting *Cost Component* of TEEM [33]): The positive relation of individual PTS on individual salaries will be stronger for high performers when compared to low ones (Level 1), and this relationship will be found in teams with large standard deviations in salary, but not in those with high average salaries (Level 2). Hypothesis 4 (supporting *Cost Component* of TEEM [33]): The positive relation of individual EFF on individual salaries will be stronger for high performers when compared to low ones (Level 1), and this relationship will be found in teams with large standard deviations in salary, but not in those with high average salaries (Level 2).

## Materials and methods

### Data collection

NBA data comprised accumulated information on the players’ salaries and statistics across an extended period of 11 years starting from the 2005/6 season to the 2015/16 season.^1^ Individual annual salaries were obtained from the player salary data provided by ESPN (http://www.espn.com/nba/salaries). Individual stats were obtained from the data provided on the NBA homepage (http://stats.nba.com). First, 779 players for whom no individual salary records could be found were deleted from the dataset (5001 to 4222 players; Table 1). Mean and standard deviation of the salary in each team by season were calculated based on the 4222 cases. The second round of data cleaning resulted in 3247 cases after deleting 975 cases in which the individual salaries for a current season could not be matched with the previous season’s individual stats.

**Table 1:** Number of Cases, Mean, and Standard Deviation of Salary by Season

Season (2000s)	Raw data (*n*)	1st DC (*n*)	2nd DC (*n*)	MSES (million $)	SDSES (million $)
15–16	476	388	298	4.97	5.23
14–15	493	399	306	4.57	4.79
13–14	482	375	294	4.57	5.00
12–13	468	370	298	4.67	4.76
11–12	478	395	300	4.54	4.69
10–11	452	380	283	4.80	4.73
09–10	441	380	291	4.83	4.82
08–09	445	381	295	5.06	4.88
07–08	451	382	283	4.56	4.60
06–07	458	390	307	4.20	4.16
05–06	357	382	292	4.11	3.95
Total	5001	4222	3247	50.88	51.61
*M*	454.63	383.81	295.18	4.62	4.69

### Methods and procedures

NBA players were nested within 30 teams. This hierarchical relationship is appropriate for multilevel modelling. The standard deviation of the salary in each team, the Level 2 variable, is useful in testing group effects (*Cost Component* or *Value Component*). Generally, we expect high-salaried players to perform better than low-salaried ones. If a team with larger standard deviations in salary for 11 years shows greater gaps, differences in individual performance among members is large. In contrast, teams with low salary deviations have small performance gaps between players. Therefore, the standard deviation of salary is more appropriate than the mean salary of teams in testing the presence of a group effect that arises when performance levels of team members vary.

Individual salaries in each season (e.g. 15–16 season) were influenced by the individual stats (e.g. EFF and PTS) from the previous season (e.g. 14–15 season). Performance Level (PL) was determined based on the mean salary (4.62 million dollars) of the 4,222 players analysed after the first round of data cleaning (Table 1). Players who earned less and more than the mean were categorised as low and high performers, respectively. PL was determined by the players’ salary in the previous season. As seen in Table 3, the Spearman’s rho (*r* = 0.65, *p* < 0.001) of the PL determined by the salaries in the previous and current seasons was not high, and there was not the problem of multicollinearity. Therefore, PL was appropriate as a performance index to predict the salary in the current season.

Level 1 equations in the random-coefficient and condition models were constructed bearing this in mind (Table 2). Level 2 data, based on the first round of data cleaning, were combined statistics for mean salary (i.e. M_slope_ and TM) and for standard deviation of the salary in each team (i.e. SD_slope_ and TSD). As there were 11 mean salaries (1 for every season included in the analysis), the x-axis represented the seasons, and the y-axis represented the corresponding mean salaries. The slope of the regression line was M_slope_. Similarly, for the standard deviation of the salary for each team, the slope across 11 seasons was SD_slope_. This implies that teams with greater M_slope_ had steadily increasing mean salaries and teams with increased SD_slope_ had increasing standard deviations, both over 11 seasons. The total mean across 11 seasons was calculated for the mean salaries, and the same was calculated for the standard deviations of the salary for each team. Considering this, Level 2 analyses in the random-coefficient and conditional models are also shown in Table 2.

**Table 2:** Analysis Models

Model	Level	Equations
Random-coefficient model	1	$$\begin{aligned} \left( {{\text{Salary}}_{{\text{current season}}} } \right)_{{{\text{ij}}}} = & \, \beta_{{0{\text{j}}}} + \, \beta_{{{\text{1j}}}} \left( {{\text{PL}}} \right)_{{{\text{ij}}}} + \, \beta_{{{\text{2j}}}} \left( {{\text{Statistics}}_{{\text{previous season}}} } \right)_{{{\text{ij}}}} \\ & + \, \beta_{{{\text{3j}}}} \left( {{\text{Statistics}}_{{\text{previous season}}} \times {\text{ PL}}} \right)_{{{\text{ij}}}} + {\text{ r}}_{{{\text{ij}}}} \\ \end{aligned}$$
	2	$\beta_{{0{\text{j}}}} = \, \gamma_{00} + {\text{ U}}_{{0{\text{j}}}} , \, \beta_{{{\text{1j}}}} = \, \gamma_{{{1}0}} + {\text{ U}}_{{{\text{1j}}}} , \, \beta_{{{\text{2j}}}} = \, \gamma_{{{2}0}} + {\text{ U}}_{{{\text{2j}}}} , \, \beta_{{{\text{3j}}}} = \, \gamma_{{{3}0}} + {\text{ U}}_{{{\text{3j}}}}$
Conditional model	1	$$\begin{aligned} \left( {{\text{Salary}}_{{\text{current season}}} } \right)_{{{\text{ij}}}} = & \, \beta_{{0{\text{j}}}} + \, \beta_{{{\text{1j}}}} \left( {{\text{PL}}} \right)_{{{\text{ij}}}} + \, \beta_{{{\text{2j}}}} \left( {{\text{Statistics}}_{{\text{previous season}}} } \right)_{{{\text{ij}}}} \\ & + \, \beta_{{{\text{3j}}}} \left( {{\text{Statistics}}_{{\text{previous season}}} \times {\text{ PL}}} \right)_{{{\text{ij}}}} + {\text{ r}}_{{{\text{ij}}}} \\ \end{aligned}$$
	2	$$\begin{gathered} \beta_{{0{\text{j}}}} = \, \gamma_{00} + \, \gamma_{{0{1}}} \left( {{\text{M}}_{{{\text{slope}}}} {\text{or SD}}_{{{\text{slope}}}} } \right)_{{\text{j}}} + \, \gamma_{{0{2}}} \left( {\text{TM or TSD}} \right)_{{\text{j}}} + {\text{ U}}_{{0{\text{j}}}} \hfill \\ \beta_{{{\text{1j}}}} = \, \gamma_{{{1}0}} + \, \gamma_{{{11}}} \left( {{\text{M}}_{{{\text{slope}}}} {\text{or SD}}_{{{\text{slope}}}} } \right)_{{\text{j}}} + \, \gamma_{{{12}}} \left( {\text{TM or TSD}} \right)_{{\text{j}}} + {\text{ U}}_{{{\text{1j}}}} \hfill \\ \beta_{{{\text{2j}}}} = \, \gamma_{{{2}0}} + \, \gamma_{{{21}}} \left( {{\text{M}}_{{{\text{slope}}}} {\text{or SD}}_{{{\text{slope}}}} } \right)_{{\text{j}}} + \, \gamma_{{{22}}} \left( {\text{TM or TSD}} \right)_{{\text{j}}} + {\text{ U}}_{{{\text{2j}}}} \hfill \\ \beta_{{{\text{3j}}}} = \, \gamma_{{{3}0}} + \, \gamma_{{{31}}} \left( {{\text{M}}_{{{\text{slope}}}} {\text{or SD}}_{{{\text{slope}}}} } \right)_{{\text{j}}} + \, \gamma_{{{32}}} \left( {\text{TM or TSD}} \right)_{{\text{j}}} + {\text{ U}}_{{{\text{3j}}}} \hfill \\ \end{gathered}$$

If, in the conditional model shown in Table 2, γ_31_ or γ_32_ are not statistically significant for M_slope_ and TM, but only for SD_slope_ or TSD, the effect of the previous season’s individual stats on the current season’s individual salaries differs based on the levels of the players’ individual performance (high or low). Thus, the effect is influenced by Level 2 variables SD_slope_ or TSD. The combined statistics for standard deviations of salary (SD_slope_ and TSD) reflect the gaps among the team members’ levels of performance and signifies that the effects of individual stats on individual salaries differ based on the team members’ level of performance. Therefore, a three-way interaction graph can determine the degree of improvement in individual performance based on the team members’ levels of performance (high or low). For instance, when the social compensation effect is present, the high performers in a team with large performance gaps (a team with large SD_slope_ or TSD) will earn higher salaries even if their performance stats are the same as high performers with low performance gaps (a team with small SD_slope_ or TSD). In contrast, when the Köhler effect is present, the low performers in a team with large performance gaps (a team with large SD_slope_ or TSD) will earn higher salaries, even if their performance stats are the same as low performers with low performance gaps (a team with small SD_slope_ or TSD). Therefore, if γ_31_ or γ_32_ are statistically significant for the combined statistics of standard deviations of salary, contrary to the combined statistics of mean salary, it is possible to distinguish whether the group effect is because of the improvement in the performance of high (social compensation) or low performers (Köhler effect). To examine this, HLM 7.0 was used for analysis, correlation and descriptive statistics were analysed using the SPSS statistical program, and the significance level was set at 0.05.

## Results

### Correlation and descriptive statistics

As PL is categorical data, the correlation between PL and salary was analysed using Spearman’s rho, and the continuous variables—salary, PTS, and EFF—were analysed using Pearson’s correlation coefficients in Table 3. The correlations between salary and PL, PTS, and EFF were significant at 0.65 (*p* < 0.001), 0.59 (*p* < 0.001), and 0.61 (*p* < 0.001), respectively. There was no multicollinearity problem because there was no high correlation. However, the correlation between PTS and EFF was very high at 0.93 (*p* < 0.001). Therefore, it is desirable to insert these two variables separately, as there was a multicollinearity problem in this context. Since there was a positive relationship between Salary and stats (PTS, EFF), convergent validity was considered secured. Table 4 shows the descriptive statistics for levels 1 and 2.

**Table 3:** Correlation Matrix

	Salary	PL	PTS	EFF
Salary	1			
PL	0.65***	1		
PTS	0.59***	0.43***	1	
EFF	0.61***	0.45***	0.93***	1

***p < .001

**Table 4:** Descriptive Statistics for Levels 1 and 2

Level 1 descriptive statistics (*N* = 3247)					Level 2 descriptive statistics (*N* = 30)		
Variable Name	Skewness	Kurtosis	*M*	*SD*	Variable Name	*M*	*SD*
Salary	1.38	1.59	5.66	5.17	*M*_*slope*_	0.05	0.16
PTS	0.82	0.44	6.63	4.70	TM	4.72	0.59
EFF	0.64	− 0.29	7.39	4.92	SD_*slope*_	0.07	0.20
					TSD	4.58	0.90

### Group effect analysis

The random-coefficient model in Table 5 shows that *γ*_*00*_ was 5.69 (*t* (29) = 48.08, *p* = 0.001), indicating that the mean salary of all participants at the individual level was similar to that shown in Table 3 (i.e. 5.66). At Level 1, after the performance level (PL), individual salary_current season_, EFF_previous season_, and EFF_previous season_ × PL was entered, the effect on individual salary_current season_ was examined. The results presented a positive fixed effect of EFF_previous season_ (β = 0.28, *t* (29) = 13.85, *p* = 0.001) and PL (β = 3.15, *t* (29) = 9.52, *p* = 0.001) on individual salary_current season_ (see Table 5). Therefore, players with higher EFF_previous season_ and PL earned higher salaries.

**Table 5:** Hierarchical Linear Model Analysis Results for Individual Salary_current season_ and EFF

Type		Random-coefficient model		Conditional model	
		*β*	SE	*β*	SE
*Fixed effect*					
*Intercept, β*_*0*_	Intercept, *γ*_*00*_	5.69***	0.11	5.69***	0.06
	SD_*slope*,_ *γ*_*01*_			0.19	0.36
	*TSD*, *γ*_*02*_			0.61***	0.07
*EFF*_*previous season*_*, β*_*1*_	Intercept, *γ*_*10*_	0.28***	0.02	2.86***	0.02
	SD_*slope*_, *γ*_*11*_			0.05	0.11
	*TSD*, *γ*_*12*_			0.01	0.02
*PL, β*_*2*_	Intercept, *γ*_*20*_	3.15***	0.33	3.13***	0.33
	SD_*slope*_, *γ*_*21*_			3.23	1.88
	*TSD*, *γ*_*22*_			− 0.26	0.39
*EFF*_*previous season*_ × *PL, β*_*3*_	Intercept, *γ*_*30*_	0.23***	0.03	0.23***	0.03
	SD_*slope*_, *γ*_*31*_			− 0.25	0.19
	*TSD*, *γ*_*32*_			0.10*	0.04
*Random effect*					
Level 1, *r*		11.28		11.24	
Level 2,* u*_*0*_		0.31***		0.02	
Level 2,* u*_*1*_		0.00		0.00	
Level 2,* u*_*2*_		1.40*		1.40*	
Level 2,* u*_*3*_		0.01*		0.01	
Total variance (i.e. ICC)		13.02 (13.3)		12.68 (11.3)	

^*^p < .05

^***^p < .001

The intra class correlation (ICC) in the random-coefficient model was 13.3%, suggesting that, of the total amount of variance in individual salary_current season,_ the proportion explained at the individual level (Level 1) by the three independent variables (PL, EFF_previous season_, EFF_previous season_ × PL) was 86.7% (11.28/13.02 × 100), and the proportion explained at the team level (Level 2) was 13.3% (1.73 / 13.02 × 100). As *u*_*3*_ at 0.018 (*χ*^2^ (29) = 46.69, *p* = 0.020) was significant for the random effects in the random-coefficient model, the slope of the interaction variable (EFF_previous season_ × PL) on individual salary_current season_ was different for each of the 30 NBA teams, meaning that there were significant parts that could be explained by the Level 2 variables. Therefore, the conditional model analysis was conducted after entering the Level-2 variables, SD_Slope_ and TSD.

**Fig. 2:**
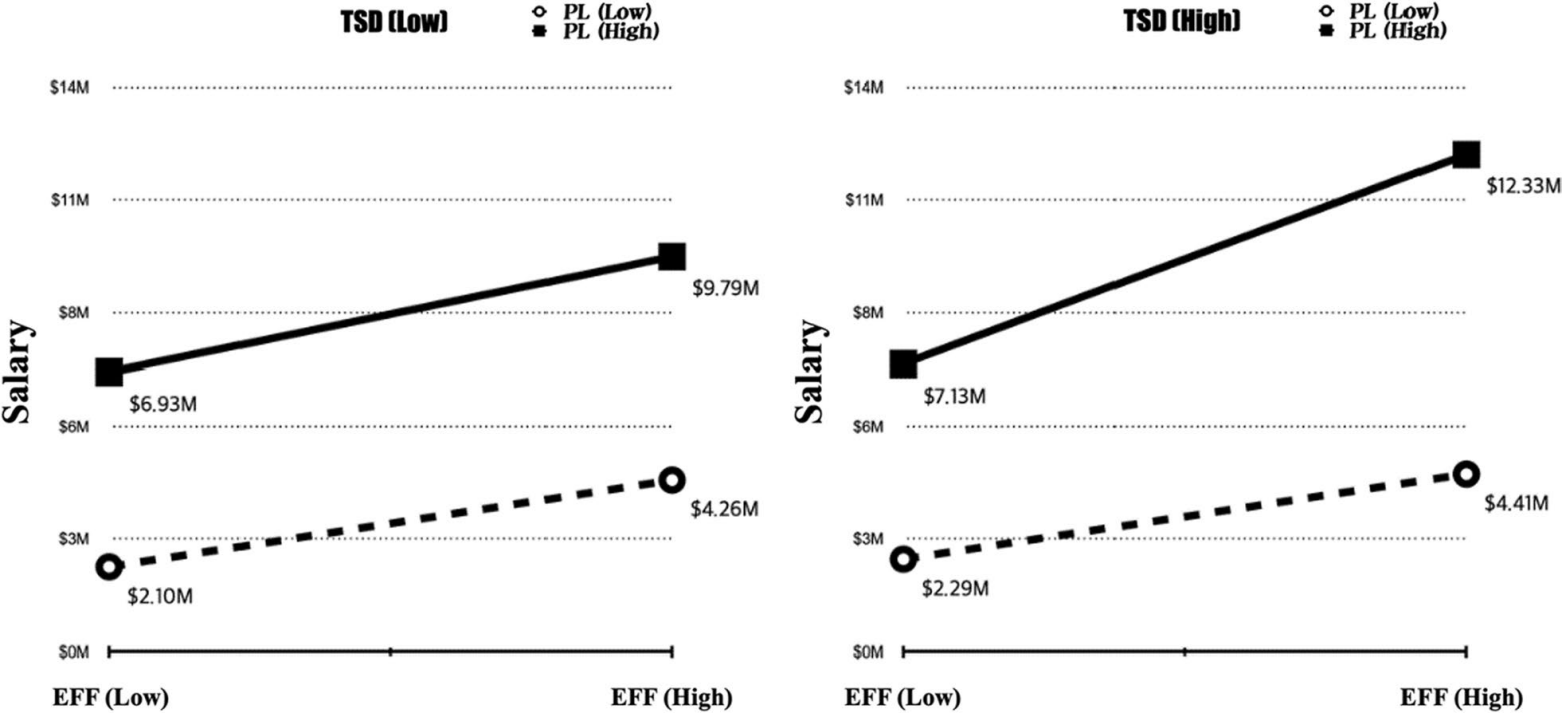
Three-way interaction graph. *Note* PL = performance level. EFF = efficiency. TSD = total mean across 11 years of standard deviation of salary for each team

The results showed that for the fixed effects in the conditional model, *γ*_*32*_ at 0.10 (*t* (29) = 2.49, *p* = 0.019) was significant. Therefore, the slope of the interaction variable EFF_previous season_ × PL was different for each of the 30 NBA teams. This difference was affected by a level-2 variable, namely TSD. To interpret this effect, the three-way interaction was graphed (Fig. 2) after identifying the top five teams with the largest TSD (high TSD group) and the bottom five teams (low TSD group).

In Fig. 2, the high performers, while selecting teams with greater TSD, that is, teams with greater variance among the team members’ performance, showed a stronger positive relationship between EFF and individual salary, compared to that while selecting teams with smaller TSD. This implies that the high performers, while selecting teams with larger performance gaps among team members, earned a higher salary than while selecting teams with lower performance gaps among team members. Interestingly, in the conditional model, where the M_slope_ and TM were entered, neither *γ*_*31*_ nor *γ*_*32*_ were significant. Therefore, assuming that salary is an objective measure of individual performance, the results can be interpreted to mean that social compensation, rather than the Köhler effect, was supported as the performance of high performers increase in NBA teams where the variance in PL among the members is large.

In Level 1 of the random-coefficient model, the PL explaining individual salary_current season_, PTS_previous season_, and PTS_previous season_ × PL was entered in order to examine the effect on individual salary_current season_. The results indicated that, for fixed effects, PTS_previous season_ (β = 0.30, *t* (29) = 13.68,* p* = 0.001) and PL (β = 3.71, *t* (29) = 12.02, *p* = 0.001) positively affected individual salary_current season_ (see Table 6). Therefore, players with higher PTS_previous season_ and performance earned higher salaries. For random effect, the ICC was 11.7%, meaning that of the total variance in individual salary_current season_ (i.e. 12.97), predicted by three independent variables (PL, PTS_previous season_, PTS_previous season_ × PL), 88.3% (11.46 /12.97 × 100) was explained at the individual level (Level 1) and 11.7% (1.51 / 12.97 × 100) was explained at the team level (Level 2). As *u*_*3*_ at 0.02 (*χ*^2^ (29) = 38.86, *p* = 0.104) was not significant, conditional model analysis was not conducted.

**Table 6:** Hierarchical Linear Model Analysis Results for Individual Salary_current season_ and PTS

Type		Random-coefficient model	
		*β*	SE
*Fixed effect*			
*Intercept, β*_*0*_	Intercept, *γ*_*00*_	5.69***	0.11
	SD_*slope*,_ *γ*_*01*_		
	*TSD*, *γ*_*02*_		
*PTS*_*previous season*_*, β*_*1*_	Intercept, *γ*_*10*_	0.30***	0.02
	SD_*slope*_, *γ*_*11*_		
	*TSD*, *γ*_*12*_		
*PL, β*_*2*_	Intercept, *γ*_*20*_	3.71***	0.31
	SD_*slope*_, *γ*_*21*_		
	*TSD*, *γ*_*22*_		
*PTS*_*previous season*_ × *PL, β*_*3*_	Intercept, *γ*_*30*_	0.21***	0.04
	SD_*slope*_, *γ*_*31*_		
	*TSD*, *γ*_*32*_		
*Random effect*			
Level 1, *r*		11.46	
Level 2,* u*_*0*_		0.31***	
Level 2,* u*_*1*_		0.00	
Level 2,* u*_*2*_		1.19	
Level 2,* u*_*3*_		0.02	
Total variance (i.e. ICC)		12.97 (11.7)	

***p < .001

## Discussion

### Statistical implications

Multilevel modelling is a method of analysing hierarchically structured data, such as when individuals belong to specific social organisations, which is useful in explaining motivation gain in groups [38, 39]. Myers and Feltz [39] suggested that multilevel models like HLM, are needed in order to examine the effect of team-level variables on individual-level variables in sports. The multilevel statistical technique that was proposed in order to confirm motivation gain in previous studies [38, 39] was applied in this study.

The players who were excluded from the first round of data cleaning in this study likely did not complete the season because of factors like injury or trade. In the second round of data cleaning, data from 975 individuals violating the assumption that the individual salary is affected by the previous season’s performance indicator were deleted. In the case of star rookies, although their current season salary data exist, the previous season individual stats do not. Therefore, the second round of data cleaning helped remove data from some players who outperformed their low salaries (e.g. star rookies). In conclusion, conducting Level 1 analysis after removing 1754 cases through the two data cleaning procedures was a means to increase the validity and reliability of the results.

Although the Level 1 data were obtained after two rounds of data cleaning, Level 2 were calculated based on the data drawn after the first round of data cleaning. The reason is that, when Level 2 variables (M_slope_, TM, SD_slope_, TSD) are calculated from the data drawn from after the second round of data cleaning, the group effect may not be represented adequately. While it would be helpful to remove the super-rookie bias to increase the statistical power of our results, in the real NBA, the presence of a star rookie on a team creates motivation gains, so we used the data from the second round of data cleaning to calculate the Lever 2 variable.

If a team has a player with an elevated performance despite a low salary (or a high salary), like a star rookie (or a high quality player despite being a low performer if they are on a long contract towards the end of their career), γ_31_ or γ_32_ is significant when Level 2 variables, M_slope_ and TM, are entered. Interpreting directionality, low performers (star rookies who were categorised as low-performing because their salaries were low, even if they actually performed exceptionally well) should tend to show a stronger relationship between individual stats and performance in a team with low M_slope_ or TM (or high performers (high quality players who were categorised as high-performing because their salaries were high, even if they actually performed exceptionally low) should tend to show a weaker relationship between individual stats and performance in a team with high M_slope_ or TM). However, this study did not find a significant γ_31_ or γ_32_ when M_slope_ or TM was entered. Therefore, the issues of validity and reliability owing to the players who outperform (or underperform) their salaries are expected to be minimal. Lastly, because of the analysis in Level 1, the random-coefficient model, EFF_previous season_, PTS_previous season, and_ PL_previous seaso_ have a statistically significant positive effect on salary_current season_, which secures predictive validity.

### Theoretical implications

Research on the group effect has focused primarily on the aspect of effort loss [31, 40]. Researchers have theorized various causes of motivation losses among individuals when working in groups compared to working alone, represented by social loafing [8], and free riding [30, 34]. It seems that dispensability of individual contributions is not a case in the NBA, since there is a clear common goal of winning among members and a clear reward for winning (prize money or salary increase), therefore no loss of motivation is observed by the *Expectancy Component* of TEEM.

This study found that motivation gain existed in high performers, which can be interpreted as support for social compensation. Social compensation is induced when a high-performing individual among group members thinks they need more of their own effort because they expect other members to perform poorly [5]. A team with a large deviation in the salary of its members is one in which high performers and low performers coexist. In such cases, motivation gain is expected to be induced as the high performers expect poor performance from the low performers. In other words, by the *Cost Component* of TEEM, the high performers have a higher effort expenditure because they believe the low performer’s ability and effort will be insufficient in achieving the common goal.

Motivational gains were consistently observed in conditions of high indispensability, whether it is a conjunctive task, where the weakest member determines the team performance; a disjunctive task [41], where the strongest member determines the team performance, or where competing later or last during sequential teamwork (e.g., the last runner in a relay race) determines the team performance [27, 42, 43]. Since the NBA’s regular season consists of 82 games in 25 weeks, it is more reasonable to explain motivation gains with the *Cost Component* rather than the *Expectancy Component,* which assumes a disjunctive task condition where every game is won or lost by a high performer.

When performance evaluations cannot be made, team members can ‘hide in the crowd’ [44], and may not recognize the need to work hard if they are not praised or criticized for their performance [45]. Torka et al. [27] argued that if team members cannot expect to earn additional benefits (e.g., credit or praise), the evaluation is unlikely to result in an effort gain. NBA players are evaluated by fans and the media, but the motivation gains in the cost component of the team’s high performers were found significant in this study. Therefore, it may be a good strategy to sign additional option contracts, based on high performers’ statistics to enhance their effort expenditure (e.g., option contracts contain benefits based on statistics, where high-performing centers are incentivized with more than regular season rebounds, and guards are rewarded based on assists, and so forth.)

Since indispensability can be realized by assigning unique tasks to team members, researchers [46, 47] divided the main task into subtasks for individual team members, to induce effort gains in team members. To increase the NBA’s low performer effort expenditure, it would be helpful to customize training based on game situations with high indispensability. For example, if you have a player with poor dribbling and speed, but a good 3-point shot, you can have him repeatedly drill a pattern of making 3-pointers in critical situations, this makes it more likely to trigger effort gain because he’s been given a task that only he can perform in situations of high indispensability.

Emich [48], using 3-player basketball teams, showed that on offense, the higher one’s confidence in one’s teammates, the more that a player will pass the ball to those who can score rather than try to score themselves. However, on defence, the higher one’s confidence in their teammates’ abilities, the more effort the player puts into the task because the weakest link on defence can dictate the entire team’s performance. In defence, high performers can induce motivation gain to compensate for the lack of lower performers. Therefore, social compensation will very likely appear in defence-related statistics. While PTS is a statistic that represents offense, EFF is a statistic that also considers defences such as rebounds, steals, and blocks. Thus, social compensation may exist in EFF, not PTS, as shown in the results of this study.

### Limitations and future research

The dataset implies a three-level model if we consider that the repeated measures data are nested within individuals and individuals are nested within teams. However, while analysing the three-level model in HLM, the row data are excluded because of constraints to meet statistical assumptions. In the three-level model, level 1 is repeat measurement data. Therefore, all players must have the same number of seasons and be on the same team. Therefore, a two-level model was used in this study.

Hüffmeier et al. [42] noted that a feeling of belonging and cohesion developed in sport field conditions, where members know each other well and have worked together for a long time, where it is more likely to trigger motivation gains in a field study than in a laboratory condition. Since various psychological factors, such as leadership, as well as the relationship and cohesion among members, can affect motivation, this study divided the performance level of members by individual annual salary rather than statistics (e.g., EFF, PTS). This is because we believe it is difficult to evaluate the relationship and cohesion of teammates and leaders through individual statistics, but individual salaries can be used to evaluate not only a player’s objective performance, but also other factors that cannot be measured through statistics (e.g., leadership, character, etc.). Future researchers will greatly benefit from examining differences in motivation gains based on relationships between members (e.g., very close friends versus general group) and participants’ internal factors through laboratory studies.

Previous studies [1–5] have looked at motivation gain from the perspective of perceived indispensability based on CEM. In this study, only the first contingency, namely ‘the individual and team performance’, was considered among the three contingencies of perceived indispensability as it was conducted based only on player salaries and personal records. Future studies should investigate the second contingency, namely ‘the team’s performance and the resulting team outcomes (e.g. players from teams that ranked higher in the league in the previous season will have an effort gain when compared to players from teams that ranked lower)’ and the third contingency, ‘the team’s outcomes and the outcomes they individually receive (e.g. playoffs are more valuable competition than regular leagues, so player effort gains may exist in playoffs)’.

According to the *Expectancy Component* of TEEM, effort expenditures can take place in anyone, regardless of whether they are high or low performers, in relay races or field events or swimming events, there is an effort gain in the fourth runner under conditions of high indispensability [1–6]. In other words, a situation with a high degree of indispensability can elicit higher performance from lower performers. Many laboratory setting studies [31, 49, 50] had similar results as in the sports settings, and it is expected that in the NBA or MLB, there will also be an improvement in the performance of low performers if there is a high degree of indispensability. In the playoffs of NBA and MLB, top teams, who likely have similar abilities, compete against each other and the performance of high performers among the teams should be similar, unlike in the regular season. In a situation where the high performer’s performance level is similar to the low performer’s, it is more likely that the low performers need to perform better for the team to win (a situation with high indispensability for low performers), so motivation gains are expected among low performers. Future research will need to verify the low performers’ effort expenditures owed to indispensability, based on playoff data from the NBA and MLB.

## Conclusions

This study tested the motivation gains in groups results from *Value Component* or *Cost Component* of TEEM by examining scaled individual salaries of the NBA players, utilising indices related to player statistics gathered over a decade. To test motivation gain, this study used 11-year NBA statistical data of 3247 players by applying HLM. The results show that social compensation, rather than the Köhler effect, caused a gain in motivation. Our results can be used for season decisions, such as personnel choices made by general managers and for the enhancement of coaching strategies, ultimately improving team morale and performance.

## Data Availability

Individual annual salaries were obtained from the player salary data provided by ESPN (http://www.espn.com/nba/salaries). Individual stats were obtained from the data provided on the NBA homepage (http://stats.nba.com).

## Abbreviations

NBA: National basketball association
CEM: Collective effort model
NFL: National football league
MLB: Major league baseball
PTS: Points
EFF: Efficiency
HLM: Hierarchical linear modelling
PL: Performance level
TM: Total mean across 11 years of mean salary for each team
TSD: Total mean across 11 years of standard deviation of salary for each team
ICC: Intra class correlation

## Footnotes

1 Data collection for this study started in 2017 and was completed in 2018, and after 2019 were not collected due to the COVID-19 pandemic.
